# Knowledge, Perception, and Willingness to Practice Dental Public Health Among Dental Students in Riyadh, Saudi Arabia: A Cross-Sectional Study

**DOI:** 10.7759/cureus.89314

**Published:** 2025-08-04

**Authors:** Salwa A Alsadhan, Hoda Abdellatif, Sultan Almalki, Albandary H Aljameel

**Affiliations:** 1 Department of Periodontics and Community Dentistry, College of Dentistry, King Saud University, Riyadh, SAU; 2 Department of Dental Public Health, College of Dentistry, Texas A&M University, Dallas, USA; 3 Department of Preventive Dental Sciences, Prince Sattam Bin Abdulaziz University, Al-Kharj, SAU

**Keywords:** community dentistry, dental education, dental public health, dentistry, dph

## Abstract

Background

This study aimed to explore the awareness, perception, and willingness of dental students toward practicing dental public health (DPH) as an extramural community activity.

Methodology

A self-administered, closed-ended questionnaire made up of 20 items was developed. Items on awareness, perception, and willingness to practice DPH were collected using a five-point Likert scale, where lower scores reflected a more positive response, and higher scores indicated a more negative one. The questionnaire was distributed as hard copies to first- to fifth-year dental students, as well as interns. The students were from the colleges of dentistry at King Saud University, Princes Nourah Bint Abdulrahman University (female-only college) (PNU), Riyadh Elm University, and Prince Sattam Bin Abdulaziz University (male-only college).

Results

A total of 1,186 students from different dental schools participated in the study. The participants perceived DPH more positively (1.85 ± 0.59 SD); however, their awareness (2.17 ± 0.70 SD) and willingness to practice (2.24 ± 0.57 SD) DPH activities were lower. Findings also showed that females and participants from PNU had, on average, more awareness, better perception, and stronger willingness to practice DPH compared to their male counterparts. Additionally, interns had, on average, more awareness about DPH.

Conclusions

It is recommended to integrate mandatory community-based rotations into the dental curriculum to provide students with firsthand experience in underserved settings and enhance their understanding of public health needs. In addition, it is recommended to enhance non-dental service-learning opportunities to foster cultural competence and social responsibility through engagement with diverse communities beyond traditional clinical settings and offering elective tracks in DPH to support students with a specific interest in public health and encourage future specialization in this major specialty area.

## Introduction

Dental public health (DPH), according to the American Association of Public Health Dentistry, is defined as the discipline and art of preventing and controlling dental diseases as well as promoting dental health through organized community efforts [1]. DPH professionals can advance oral health by assessing the oral health status at the community level; informing, educating, and empowering people about health issues; and developing and implementing policies and plans that support individual and community health efforts [2]. A report on the global burden of disease published in 2019 noted that oral disorders affect more than 44.5% of the global population [3]. These disorders can lead to pain, school absenteeism, reduced work performance, and reduced overall quality of life [4]. Oral health was recorded by the World Health Organization as one of the top 10 standards for human health, and in its analysis report, it suggested that oral diseases have become a crucial factor in human quality of life and a global public health burden [5,6].

A systematic analysis of the Global Burden of Disease study reported that oral health conditions have remained a substantial population health challenge in the last 30 years [7]. Consequently, there is a need for effective and evidence-based dental public health practices to minimize the burden of such diseases at the individual as well as at the country level. To address health disparities, it is vital to expose undergraduate dental students to the nature of health disparities, primary care dentistry, and cultural competency, among others [8]. Dental students also need interprofessional experiences to develop skills for affiliation with community dental clinics and the chance to work with culturally diverse groups during their training [8].

In the United States, the actual number of extramural clinical rotations that help in improving community responsibility skills for predoctoral dental students has grown. According to the annual American Dental Education Association (ADEA) senior survey, dental students’ extramural experience affected their ability to provide care to culturally, racially, and ethnically diverse groups [9]. In the same survey, a great percentage of the participating students agreed that access to oral health care was a “societal good and right,” and an even greater percentage agreed that ensuring and providing care to all segments of society is an ethical and professional obligation. The Annual ADEA Survey of Dental School Seniors of the 2017 graduating class found that the majority of students planned to teach and work with underserved populations at some point in their careers [10]. However, between 2017 and 2022, the interest in joining private practice immediately after graduation increased among the respondents from 48% to 53% [11].

Exposure to DPH concepts during the early years is quite significant. It can also improve a wide range of non-clinical skills such as research skills, program administration, program development, consultative services, dental health education, and professional education [9]. Therefore, future dental professionals need to be introduced to the fundamental concepts of DPH and oral epidemiology to tackle the widespread oral diseases and conditions.

A study conducted to assess the Influence of a Community-Based Dental Education (CBDE) on Practice Choices at East Carolina University found positive attitudes toward the CBDE program before and after participation [12]. In the context of Saudi Arabia, evidence suggests a very high prevalence of oral disease, mainly dental caries [13-16]. Both governmental and non-governmental bodies are working hand in hand to improve the health of citizens (including oral health), and this is stated clearly in the National Vision 2030 [17]. All the above concepts of DPH, in light of the oral health problems in Saudi Arabia, are essential to increase students’ awareness and sensitivity to DPH problems and dental public health activities. However, it is still not clear whether dental students in Saudi Arabia are prepared to contribute to this. Furthermore, dental treatment alone cannot solve this problem. An entirely different approach is now needed to tackle this global health challenge [18].

During the final year of dental schools in Saudi Arabia, courses pertaining to DPH are integrated as part of the curriculum in the College of Dentistry at King Saud University (KSU), Princess Nourah Bint Abdulrahman University (PNU), Prince Sattam Bin Abdulaziz University (PSAU), and Riyadh Elm University (REU). Students are also engaged in community-based extracurricular activities during their school years and their internship year. No studies have been found in the relevant literature to study the effect of these activities on dental graduates in Saudi Arabia, and to our knowledge, no such survey has ever been done to explore this in Saudi dental schools/colleges. This study aimed to explore the awareness, perception, and willingness of dental students toward practicing DPH as an extramural community activity.

## Materials and methods

Study design

This descriptive, cross-sectional study was conducted on a sample of dental students and interns in Riyadh, Saudi Arabia.

Questionnaire design and distribution

A self-administered, closed-ended questionnaire was created by reviewing the relevant literature. The questionnaire comprised 20 items and was structured in English (Appendices). Face validity was established through expert review by a panel of DPH specialists. Experts assessed the clarity, relevance, and domain alignment of each item, as well as the overall comprehensiveness of the questionnaire within the study’s objectives.

The participants were asked to answer three demographic questions and 17 DPH-related questions to explore the awareness, perception, and willingness to practice DPH using the Likert-type scale: 1 (strongly agree), 2 (agree), 3 (neutral), 4 (disagree), and 5 (strongly disagree). *Awareness* reflected the students’ knowledge and understanding of DPH. *Perception* encompassed the students’ attitudes, beliefs, and viewpoints regarding the importance, role, and influence of DPH within the dental profession. *Willingness* to practice referred to the students’ inclination or readiness to engage in a career or professional activities related to DPH before or after graduation. These definitions help establish a clearer conceptual foundation and support consistent interpretation throughout the study, including the survey, results, and discussion.

Before launching the questionnaire, it was pilot-tested (n = 114, male and female students from two dental schools) and evaluated for clarity of wording, readability, and layout. No adjustment was needed, and data from the pilot study were excluded from the final data analysis.

The sample utilized in this study was obtained through convenience sampling, as participants were selected based on their availability and willingness to participate. The questionnaire was distributed as hard copies, and students/interns were asked to spend around 10 minutes answering it. It was clarified that completion of the questionnaire was voluntary, and the participants had the right to stop or discontinue answering the questionnaire at any time without any consequences. No faculty member was present to avoid interference with each individual’s free will. Data collection was conducted from October 2021 to March 2022.

Study population

First to fifth-year dental students and interns were included from the colleges of dentistry at KSU, PNU (female-only college), REU, and PSAU.

Statistical analysis

All statistical analyses were performed using SPSS version 26 (IBM Corp., Armonk, NY, USA) for inferential testing and STATA (StataCorp., College Station, TX, USA) for data visualization. The study employed both descriptive and inferential methods, appropriate for a cross-sectional survey design based on Likert scale responses. Composite scores were calculated for three main domains, namely, awareness, perception, and willingness to practice DPH. Descriptive statistics (frequencies and percentages) were employed to summarize participant demographics (gender, university, and academic year). To compare the means of individual items and domain scores between male and female participants, independent-sample t-tests were used. To compare domain means across four universities and six academic levels (years 1-5 and interns), One-way analysis of variance was used. P-values are provided for each comparison, with statistical significance determined at p-values <0.05.

## Results

As shown in Table 1, there were 1,186 participants, of whom 56.79% were males and 43.21% were females. Regarding the university of the participants, 40.64% were from REU, 36.93% were from KSU, 11.8% were from PNU, and 10.62% were from PSAU. The participants’ distribution between the different levels of the dental undergraduate program was 12.06% from Year 1, 19.22% from Year 2, 16.53% from Year 3, 24.03% from Year 4, 19.65% from Year 5, and 8.51% from the Internship year. PNU had no male participants as the college only teaches female students.

**Table 1:** Descriptive statistics of the study sample. KSU = King Saud University; PNU = Princess Nourah Bint Abdulrahman University; REU = Riyadh Elm University; PSAU = Prince Sattam Bin Abdulaziz University

	Male	Female	Total
Variable	n (%)	n (%)	n
University			
KSU	228 (52.05)	210 (47.95)	438
PNU	0 (0)	140 (100)	140
REU	319 (66.18)	163 (33.82)	482
PSAU	126 (100)	0 (0)	126
Total	673 (56.75)	513 (43.25)	1,186
Year			
Year 1	66 (46.15)	77 (53.85)	143
Year 2	133 (58.33)	95 (41.67)	228
Year 3	112 (57.14)	84 (42.86)	196
Year 4	159 (55.79)	126 (44.21)	285
Year 5	133 (57.08)	100 (42.92)	233
Internship	70 (69.3)	31 (30.7)	101
Total	673 (56.79)	513 (43.21)	1,186

The means and SDs of the participants’ levels of awareness, perception, and willingness to practice DPH stratified by sex are presented in Table 2. Lower scores reflect a more positive response and higher agreement, while higher scores indicate a lower agreement. Statistically significant differences were found between males and females with regard to the majority of the questionnaire items, and on the overall domain scores (p < 0.05). Participants were asked to score their answers on a five-point Likert scale, where “1” indicated strong agreement, while “5” indicated strong disagreement. Participants perceived DPH more positively (mean = 1.85, SD = ±0.59) compared to their awareness about DPH (mean = 2.17, SD = ±0.70), and willingness to practice in DPH activities (mean = 2.24, SD = ±0.57).

**Table 2:** Means and SDs of the participants’ levels of awareness, perception, and willingness to practice DPH stratified by gender. Statistical test: independent-sample t-test. DPH = dental public health

	Variable	Male		Female		All		P-value
		Mean	SD	Mean	SD	Mean	SD	
Awareness	DPH is a specialty recognized by the American Dental Association	2.11	0.95	1.97	0.95	2.05	0.95	0.0095
	DPH incorporates prevention programs such as fluoridation, placement of sealants, and oral cancer screening to target only the underserved	2.42	1.14	2.51	1.33	2.46	1.23	0.2088
	The community dental projects constitute a part of the curriculum of the Dental Public Health Division	2.10	0.86	1.86	0.90	2	0.88	<0.0001
	Average	2.21	0.66	2.11	0.75	2.17	0.70	0.0199
Perception	There is a growing need to treat the underserved and low-income population	1.69	0.82	1.57	0.90	1.64	0.85	0.0153
	Universal access to oral healthcare is an example of social justice	1.93	0.85	1.77	0.91	1.86	0.88	0.0021
	The belief that universal access to oral healthcare is a social justice imperative will encourage me to provide care to underserved patients after I graduate	1.91	0.86	1.75	0.87	1.85	0.87	0.0017
	Health status including oral health is a matter of individual responsibility	2.03	0.97	2.01	1.04	2.02	1.00	0.7845
	Treating patients in dental school-based clinics and community health clinics helps me develop the requisite competencies for serving the underserved population	1.95	0.84	2.78	0.87	1.88	0.85	0.0009
	Average	1.90	0.51	1.78	0.65	1.85	0.59	0.0004
Willingness	Caring for the underserved in my private dental office will be a part of my professional routine	2.13	0.96	1.90	0.96	2.03	0.97	0.0001
	Working as a full-time dentist in a community dental clinic setting is an option I would consider	2.49	1.07	2.51	1.08	2.50	1.07	0.7999
	Working as a part-time dentist (<50%) in a community dental clinic setting is an option I would consider	2.35	0.99	2.11	0.95	2.25	0.98	<0.0001
	Caring for the underserved will be a part of my professional routine	2.12	0.90	1.87	0.92	2.01	0.92	<0.0001
	Participating in volunteer work will be a part of my professional routine	2.10	0.95	1.86	0.97	2.00	0.96	<0.0001
	As my career starts, volunteering my time and services to help the community will be an important aspect of my professional life	2.02	0.90	1.87	0.94	1.96	0.92	0.0035
	After my practice has been established, volunteering my time and services to help the community will be an important aspect of my professional life	2.06	0.89	1.90	0.96	1.99	0.93	0.0040
	Only after retirement, volunteering my time and services to the community will be an important aspect of my professional life	2.68	1.16	2.68	1.25	2.68	1.20	0.9302
	I would prefer to provide financial help (e.g., donations) instead participating in the above	2.64	1.15	2.85	1.17	2.73	1.16	0.0017
	Average	2.29	0.52	2.17	0.62	2.24	0.57	0.0006

**Figure 1:**
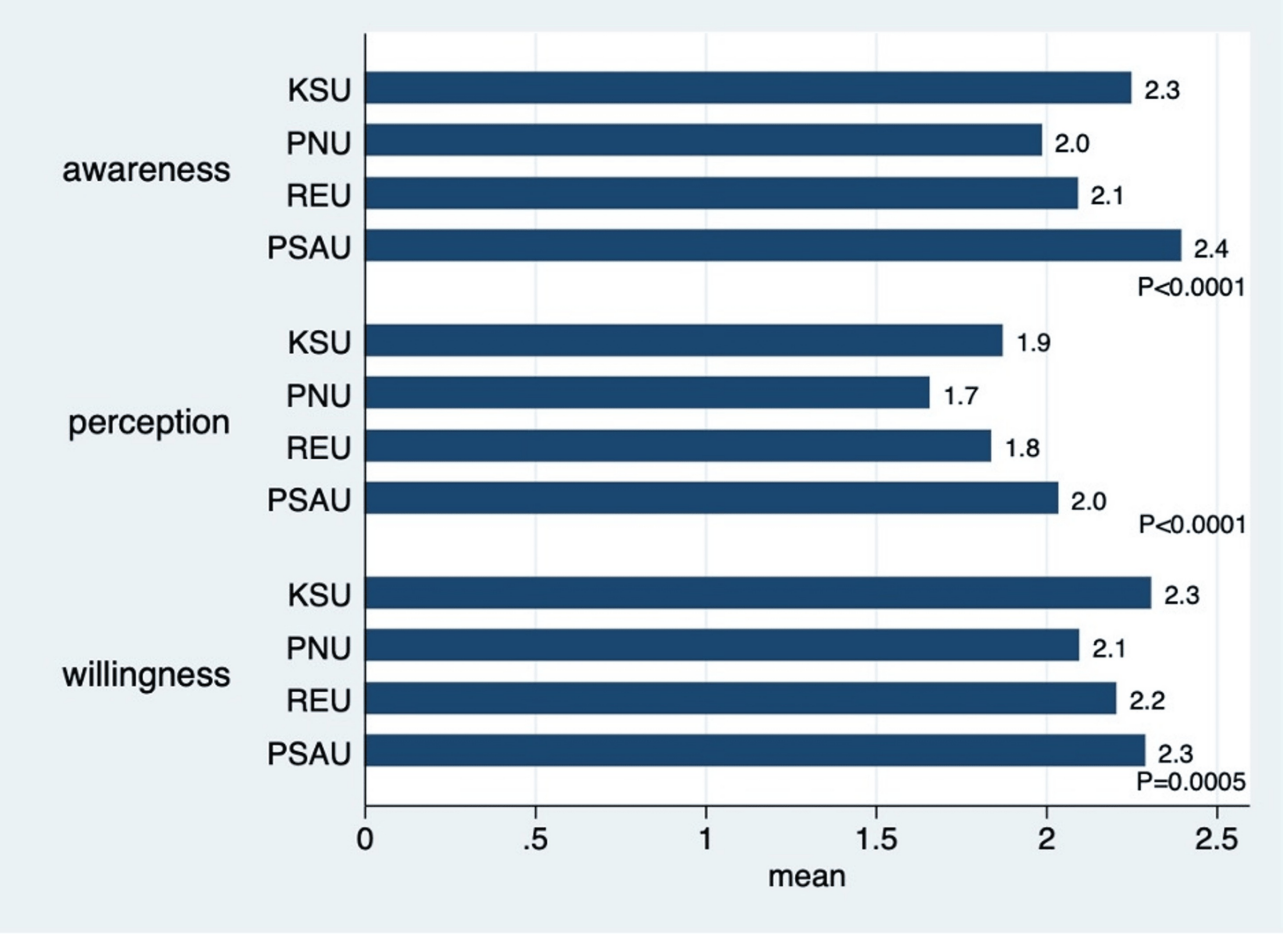
Mean scores grouped by domains and stratified by school. Statistical test: one-way analysis of variance. 1 = strongly agree; 5 = strongly disagree. KSU = King Saud University; PNU = Princess Nourah Bint Abdulrahman University; REU = Riyadh Elm University; PSAU = Prince Sattam Bin Abdulaziz University

Figure 1 shows that PNU participants (a female-only college) had, on average, more awareness, better perception, and stronger willingness toward practicing DPH, followed by REU participants. PSAU participants (a male-only college) had, on average, the lowest awareness and perception of DPH. This was represented by a lower mean of the answers to the five-point Likert scale, where “1” indicated strong agreement with the statements while “5” indicated strong disagreement.

**Figure 2:**
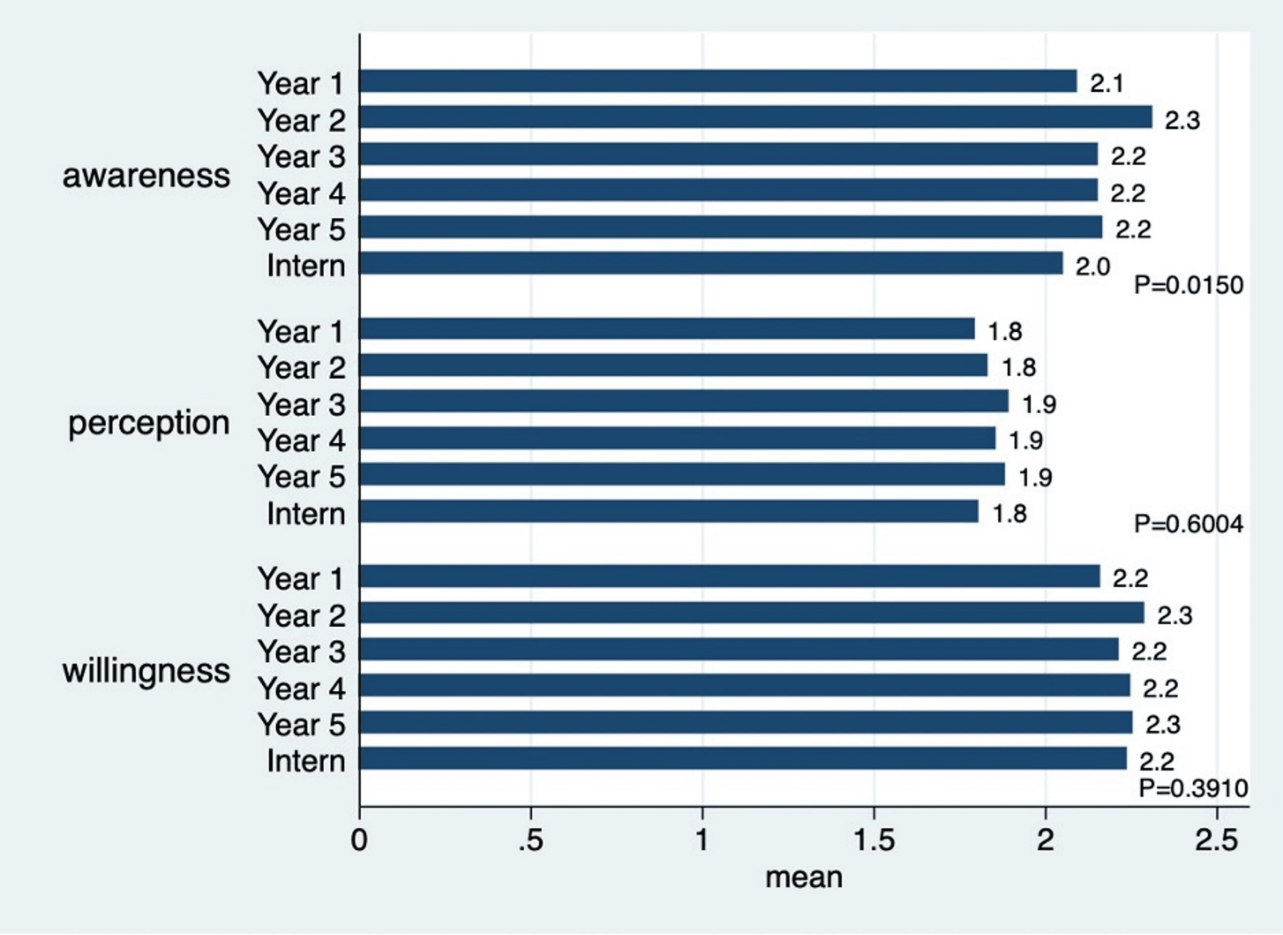
Mean scores grouped by domains and stratified by student level in the program. Statistical test: one-way analysis of variance. 1 = strongly agree; 5 = strongly disagree.

Figure 2 shows that interns had, on average, more awareness about DPH, but no statistically significant differences were found with regard to perception and willingness among participants from different academic years and interns.

Overall, the results of this study showed a positive awareness, perception, and willingness to practice DPH among the studied sample of students and interns from various dental colleges and schools in Riyadh, Saudi Arabia.

## Discussion

This study aimed to investigate the awareness and perception of dental students toward DPH, as well as their willingness to practice it as an extramural community activity. The study showed that participants perceived DPH more positively compared to their awareness and willingness to practice its activities. Results showed that female participants, on average, had more awareness, better perception, and stronger willingness to practice DPH compared to their male colleagues. This is in agreement with a study conducted to assess the attitude toward public health dentistry as a career among dental students in Odisha, India, where female students expressed a high level of interest in choosing public health dentistry as their future career in comparison to their male counterparts [19]. Likely causes for this attitude toward DPH among male students might be lower income, poorer working conditions, and significant challenges associated with the specialty to provide top oral health for widely diverse groups compared to other specialties of dentistry [19]. A study conducted in Brazil also found that women choose dentistry mainly because they like working with people, and they appear to be more concerned with the humanization of practice [20].

This study also found that PNU participants had more awareness, better perception, and stronger willingness toward practicing DPH, followed by REU participants. This might be because all PNU participants were females. Their early exposure to DPH activities might also have contributed to such findings. Interns had more awareness about practicing DPH, but no statistically significant differences were found in their perception and willingness. Female students and/or those from specific universities scored better. This could be related to variations in curriculum design, such as the integration of public health topics; differences in teaching methodologies that promote greater student engagement or critical thinking; or differing levels of exposure to community outreach initiatives, health awareness campaigns, or extracurricular programs that emphasize relevant skills and knowledge. However, no exploration of these differences was conducted in this study. Future studies might explore this area to better understand the situation and, therefore, better plan to enhance DPH training across different dental schools.

The results of this study showed a positive awareness, perception, and willingness to practice DPH among the studied sample of students and interns, which might be linked to the exposure of these students to the courses about DPH. This is in agreement with a study conducted at the University of North Carolina School of Dentistry, which also showed that dental students who had participated in the required rotations in community settings developed increased self-awareness, empathy, communication skills, and self-confidence. They were also confronted with a wide range of situations not typically encountered in dental academic settings, which enhanced their awareness of the complexities of dental care, raised complex ethical dilemmas, and heightened their sense of professional identity [21]. A study at the University of Missouri-Kansas City School of Dentistry also concluded that a significant change in a positive direction was noticed in student attitudes about volunteering in the community over the course of a dental-related program designed to enrich ethics instruction [22].

An interesting program called SCOPE (Student Community Outreach Program and Education) was developed at the University of Pittsburgh, School of Dental Medicine, to evaluate changes in dental students’ attitudes and beliefs about community service and changes in cultural competencies after participation in a two-year program of non-dental community service [23]. Their data indicated that students gained insight and experience in the following three areas: exposure to and awareness of community oral health, professional roles in the oral healthcare system, and first-time experience with delivery of care. The study provided support for the addition of a non-dental community service-learning program into the preclinical curriculum [23]. Furthermore, a study conducted in the same university by the Division of Dental Hygiene supported the finding that service learning is an effective learning strategy for increasing student awareness of underserved populations, cultural diversity, and ethical patient care. The study also suggested that service learning helped students to determine their level of interest in public health as a career choice [24].

In Saudi Arabia, many dentists work within public health settings such as city, provincial, and regional health departments; correctional facilities; nursing and long-term care facilities for the elderly and those with various disabilities; school-based health programs; and free and/or volunteer clinics. However, many do so without formal public health training [25]. This presents a complex and challenging problem that requires multi-front solutions borrowing constructs from multiple disciplines [21]. Understanding this issue outlines the importance of training dentists and exposing them to proper planning and execution of oral health programs at the public health level. This study revealed interesting findings that might be utilized to improve the existing situation and help dental schools to invest more in formal public health training.

It is important to acknowledge the limitations of the current study. One key limitation is the potential for response bias inherent in self-reported measures, such as willingness, where participants may provide socially desirable responses rather than reflecting their true intentions or behaviors. This type of social desirability bias is particularly relevant in public health research, where participants may align their answers with perceived expectations. Although the sample size was adequate to meet the study’s objectives, the fact that participants were exclusively drawn from Riyadh, a major urban center and the capital, limits the generalizability of the findings to other regions. Riyadh’s unique geographic, cultural, and socioeconomic characteristics may not represent the broader population. Future studies should consider validating the questionnaire using established psychometric tools to improve its reliability and overall quality.

Based on the findings of this study, it is recommended that a psychometric validation of the questionnaire be undertaken to strengthen the reliability and validity of the results. Future research should also consider incorporating additional variables such as socioeconomic status, urban versus rural background, career aspirations, and differences in curricular structures across institutions (e.g., differences in core competencies in DPH across institutions, differences in the required applied practice experiences in DPH, and integrative projects in the field of DPH). Furthermore, examining the influence of faculty mentorship and interdisciplinary collaboration could provide deeper insights into institutional variations. Integrating theory-driven frameworks such as the Theory of Planned Behavior and the Health Belief Model may further enhance the credibility and theoretical grounding of future studies.

## Conclusions

DPH is a unique specialty of dentistry where the focus is on the community rather than an individual, and on prevention rather than only treating existing disease. The target population is often vulnerable, with poor access to the dental care delivery system, at higher risk of developing dental disease, and with limited resources at their disposal. This study showed that the participants perceived DPH more positively; however, their awareness and willingness to practice DPH activities were lower. Hence, there is a need to extend the scope of the DPH specialty, make it more feasible, and provide appropriate training and guidance at the undergraduate level. Incorporating non-dental community service-learning programs to foster cultural competence and social responsibility and provide students with real-world experience in public health patient care into the preclinical curriculum might help address the needs and gaps in oral public health in the country.

## Figures and Tables

**Figure 3:**
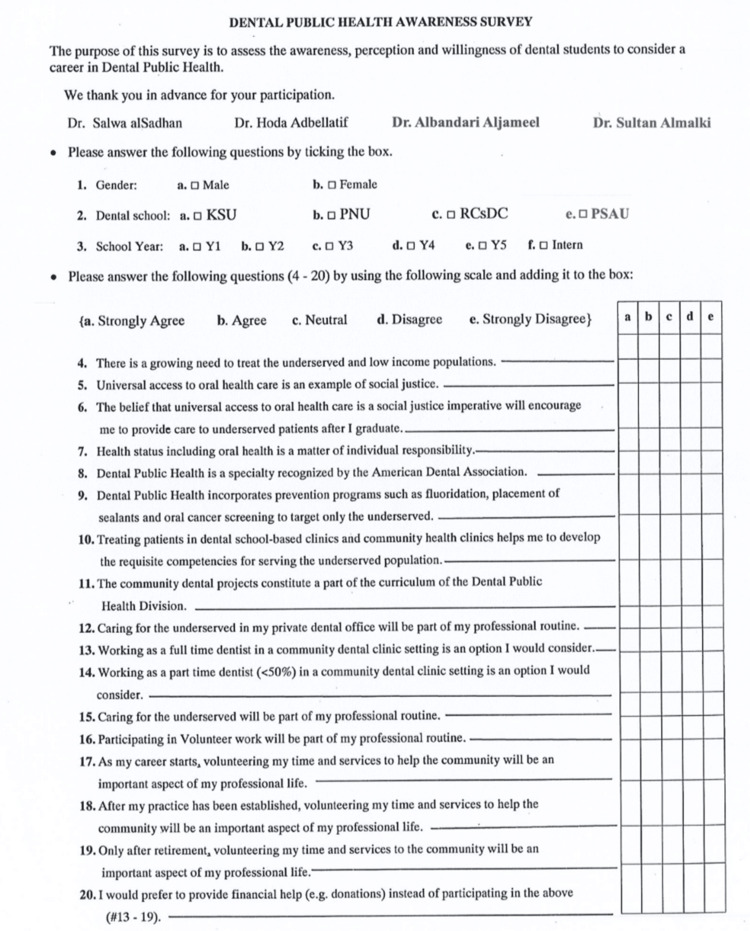
Dental Public Health questionnaire.
